# Comparative Analysis of Skeletal Muscle Transcriptional Signatures Associated With Aerobic Exercise Capacity or Response to Training in Humans and Rats

**DOI:** 10.3389/fendo.2020.591476

**Published:** 2020-10-26

**Authors:** Yildiz Kelahmetoglu, Paulo R. Jannig, Igor Cervenka, Lauren G. Koch, Steven L. Britton, Jiajia Zhou, Huating Wang, Matthew M. Robinson, K Sreekumaran Nair, Jorge L. Ruas

**Affiliations:** ^1^ Molecular and Cellular Exercise Physiology, Department of Physiology and Pharmacology, Biomedicum. Karolinska Institute, Stockholm, Sweden; ^2^ Department of Physiology and Pharmacology, The University of Toledo College of Medicine and Life Sciences, Toledo, OH, United States; ^3^ Department of Anesthesiology, University of Michigan, Ann Arbor, MI, United States; ^4^ Department of Molecular and Integrative Physiology, University of Michigan, Ann Arbor, MI, United States; ^5^ Li Ka Shing Institute of Health Sciences, Department of Orthopaedics and Traumatology, The Chinese University of Hong Kong, Hong Kong, China; ^6^ School of Biological and Population Health Sciences, College of Public Health and Human Sciences, Oregon State University, Corvallis, OR, United States; ^7^ Department of Integrative Physiology, Division of Endocrinology, Diabetes and Nutrition, Mayo Clinic, Rochester, MN, United States

**Keywords:** skeletal muscle, exercise, transcriptome (RNA-seq), aerobic capacity, response to training, human studies, rat models of exercise

## Abstract

Increasing exercise capacity promotes healthy aging and is strongly associated with lower mortality rates. In this study, we analyzed skeletal muscle transcriptomics coupled to exercise performance in humans and rats to dissect the inherent and response components of aerobic exercise capacity. Using rat models selected for intrinsic and acquired aerobic capacity, we determined that the high aerobic capacity muscle transcriptome is associated with pathways for tissue oxygenation and vascularization. Conversely, the low capacity muscle transcriptome indicated immune response and metabolic dysfunction. Low response to training was associated with an inflammatory signature and revealed a potential link to circadian rhythm. Next, we applied bioinformatics tools to predict potential secreted factors (myokines). The predicted secretome profile for exercise capacity highlighted circulatory factors involved in lipid metabolism and the exercise response secretome was associated with extracellular matrix remodelling. Lastly, we utilized human muscle mitochondrial respiration and transcriptomics data to explore molecular mediators of exercise capacity and response across species. Human transcriptome comparison highlighted epigenetic mechanisms linked to exercise capacity and the damage repair for response. Overall, our findings from this cross-species transcriptome analysis of exercise capacity and response establish a foundation for future studies on the mechanisms that link exercise and health.

## Introduction

Large-scale clinical studies reveal a strong link between low exercise capacity and a shorter lifespan and a higher probability of developing complex metabolic diseases (1, 2). Representing up to 40% of human body weight, skeletal muscle is an obvious and important target to explore the molecular connections between exercise capacity and human health (3). Adaptations to exercise include transient transcriptome changes following a bout of exercise and sustained changes in gene expression induced and maintained by training (3). These changes are orchestrated by the combinatorial activity of transcription factors, coactivators, and other signalling mediators that regulate specific gene programs to induce exercise-induced adaptations (3, 4).

Exercise capacity is a complex trait resulting from the sum of genetic and environmental factors. The former can be further divided into intrinsic (already present in the untrained state) and extrinsic (acquired) components, which are able to explain up to 50% of the total phenotypic variance observed in humans (5–7). Animal models developed for each component opened new avenues to investigate their associations with physical fitness and complex diseases. To model the intrinsic component of aerobic exercise capacity, high and low capacity runner rat lines (HCR and LCR, respectively) were derived from a heterogeneous founder population of rats (N:NIH) with breeder selection based on untrained treadmill running capacity (8). As previously shown in humans, cross-generational genetic studies revealed that these rats display aerobic capacity as a heritable trait (9), and that the LCR rats are more susceptible to a variety of diseases, particularly metabolic disorders (10). Interestingly, studies with earlier generations of HCR rats suggested that the primary driver of the high capacity phenotype is improved skeletal muscle oxygenation (11–13).

In humans and rodents alike, some individuals show clear responses to endurance training, whereas others show lower or no improvement in certain physiological traits, such as maximal oxygen consumption (10, 14). Although early studies in humans showed that this entails a genetic component, accounting for 47% of the variance in the improvement of VO_2_max (7, 15), the factors underlying responsiveness to aerobic exercise remain largely unknown. In rats, the proportion of aerobic capacity variation explained by genetic factors is around 45% (9). To model the acquired component of exercise capacity in rats, a similar breeding strategy to the HCR model was developed, but based on the high and low response to exercise training (HRT and LRT, respectively) (16). Across generations, this artificial selection for trainability yielded two lines with same baseline capacity that contrast in their response to the same endurance training program.

To date, transcriptional analysis of HCR muscles was performed by targeted approaches or using probe-based gene expression arrays and the exercise response model has not been studied as extensively (17–19). Transcriptome-wide comparison of the intrinsic and acquired capacity profiles in skeletal muscle could offer novel paths to unravel the link between exercise capacity and health. In this study, we performed a comparative analysis of skeletal muscle transcript profiles linked to specific measures of exercise performance in rats and humans. Our data identifies molecular pathways and specific genes as potential mediators of exercise capacity and trainability.

## Materials and Methods

### Animals

All animals were housed, selectively bred and experimental procedures carried out in accordance with the Institute for Laboratory Animal Research Guide for Care and Use of Laboratory Animals and in compliance with guidelines of University Committee of Use and Care of Animals at the University of Michigan.

#### Selective Breeding for Exercise Capacity Model

The models for low and high exercise capacity, LCR (low capacity runners) and HCR (high capacity runners), have been previously described in detail (8). Briefly, the rats were subjected to two-way selective breeding for untrained aerobic capacity as measured by endurance running. Starting from a genetically heterogeneous founder population (N:NIH stock), 13 pairs of the lowest and the highest running capacity rats from each sex were randomly matched for mating. Subsequently, for each generation the offspring from each family were tested for treadmill running capacity at 11 weeks old age and subjected to selection in the same manner. In this study, archived frozen gastrocnemius muscle samples taken from 3 males each from generation 33 HCR and LCR rats were used. The exercise capacity phenotype was confirmed with a treadmill exercise protocol as described (8).

#### Artificial Selection for Acquired Capacity Models

Low response trainer (LRT) and high response trainer (HRT) male rats were housed and maintained as described in detail before (16). In brief, 10–11-week-old males and females from the genetically heterogeneous N:NIH stock were subjected to incremental treadmill running test identifying their pre-training capacity (DIST1). Then each rat took on a treadmill running training program (3 days/week for 8 weeks). The program progressively increased speed by increments of 1 m/min and duration by 0.5 min in each session, starting at 10 m/min for 20 min in the 1st week and finishing at 21 m/min for 31.5 min in the 8th week. At the end of the training program, post-training exercise capacity of each rat was evaluated (DIST2) and the response to training measured as the change in maximal running distance (ΔDIST = DIST1 – DIST2). At each generation, 10 males and 10 females with the highest response were selectively bred for HRT line and separately 10 pairs with the lowest training response were bred to develop LRT line. Nearly 100 offspring per line per generation were evaluated for response to training. All training sessions and the maximal exercise capacity tests were performed on a motorized treadmill at a 15° incline.

#### Endurance Training of High and Low Responder Lines

For this study, male rats from generation 15 responder lines underwent exercise capacity testing at 10–12 weeks of age. A subset of rats then was subjected to the 8-week exercise training protocol as described above. A separate group of rats acted as baseline controls and did not undergo any training, represented as LRT and HRT groups (n = 3 per group). At the end of the training period, all rats (both trained and untrained) were tested for exercise capacity to measure exercise response as described. Trained rats are represented as LRTT and HRTT (n = 3 per group). Gastrocnemius muscles were dissected from animals 48 hours after the last exercise test, flash-frozen in liquid nitrogen and stored at -80C^◦^ for later analysis.

### RNA Sequencing

Frozen gastrocnemius muscles were pulverized using mortar-pestle. Total RNA from 50-100mg tissue powder was isolated using Trizol reagent. RNA was treated with DNAse and purified using NucleoSpin RNA II columns (Machery Nagel) and integrity was confirmed using an Agilent Bioanalyzer. RNA-sequencing was performed at GATC Biotech (Konstanz, Germany). Illumina Stranded TruSeq mRNA Library preparation kit was used with 1 ug of total RNA for the construction of sequencing libraries, which were loaded onto Illumina HiSeq 2500 High-output flow cell and sequenced in a 1 × 50 bp single read format.

### Human Training Study

Human study design is detailed in Robinson et al. (20). Briefly, the study randomly assigned young and old healthy participants to three groups: resistance training (R), high-intensity interval training (HIIT), and combined training (C). Participants trained for 12 weeks followed by repeated testing days. In this study, only the data from young participants were included in the analysis. HIIT protocol consisted of 3 days per week cycling and 2 days per week walking on a motorized treadmill. An interval session was designed to achieve 16 min of training time at a high intensity (> 90% VO_2_max). The treadmill walk was at a self-selected pace for 45 min at 70% VO_2_max. The combined training program was 5-days per week, 30 min of cycling, followed by 30 min of resistance training. The cycling at 70% VO_2_max was 20 min.

Mitochondrial respiration measurements were used as a proxy of aerobic exercise capacity. As explained in Robinson et al., mitochondria were isolated from pre- and post-training skeletal muscle biopsies and analyzed by high-resolution respirometry. Mitochondria were added to a 2mL chamber (Oxygraph-2K, Oroboros) followed by sequential additions of substrates and inhibitors. For this study, State 3 respiration through Complex I+II (Glutamate-malate-succinate) was used as mitochondrial respiration. Biopsy samples from *m. vastus lateralis* muscle were collected in rested and fasted state at 72 h post-exercise to avoid acute effects of exercise on gene abundance.

#### Bioinformatic Analysis

For RNA sequencing analysis, quality control of raw reads was determined using FastQC tool kit (http://www.bioinformatics.babraham.ac.uk/). The reads were then aligned with reference genome of *Rattus Norvegicus* (Rnor_6.0) downloaded from ENSEMBL using Tophat2 aligner (21). Alignments were assembled into transcripts using Cufflinks and list of differentially expressed genes (DEGs) was obtained by quantifying transcripts using Cuffdiff program (22).

Pathway analysis was carried out with the Ingenuity Pathway Analysis tool (QIAGEN Inc., https://www.qiagenbioinformatics.com/products/ingenuitypathway-analysis, version 8.6) and PANTHER Gene Ontology classification (23). Differentially expressed genes were analyzed for statistical overrepresentation of GO Slim Biological Processes with Fisher’s Exact test and Bonferroni correction for multiple comparisons.

Raw counts for RNA-seq dataset from Robinson et al. (20) were downloaded from NCBI (GSE97084).

For the human aerobic capacity analysis, we correlated gene expression levels at untrained state with pre-training mitochondrial respiration measurements. Since this analysis is based on only the untrained state (intrinsic aerobic capacity), participants from all three groups (HIIT, C, and R) were included for statistical power.

For human exercise response analysis, after sorting the response rates of participants based on the change in mitochondrial respiration, four participants who showed highest improvement were designated as high responders. Four participants with the lowest change in mitochondrial respiration were designated as low responders. Next, we used DESeq2 package to normalize the data as well as to identify the DEGs in pre- and post-training states of high and low human responders (24). Finally, we investigated the overlap of DEGs with same directionality of change between humans and rats for further analysis.

To explore the putative upstream regulators, we used Distant Regulatory Elements of co-regulated genes (DiRE) analysis (25). Briefly, DIRE program identifies 3 top evolutionary conserved regions (ECRs) and the promoter ECRs for each gene in the submitted dataset. After defining these regions and corresponding sequences, it feeds this information into an algorithm that searches for the known transcription factor binding motifs (TFBMs) and how enriched these are in the submitted gene set. In parallel, the algorithm uses 5000 randomly selected genes and runs the same analysis as background.

### Secretome Analysis

For the secretome prediction, we took the Emanuelsson et al. (26) protocol as a baseline for sequence-based subcellular localization prediction. We applied the following package with default settings for the pipeline (the code is available publicly at https://travis-ci.org/github/fmaguire/predict_secretome). Briefly, after converting the gene names to amino acid sequences using Uniprot, the pipeline starts with SignalP to identify proteins containing signal peptides. After that, to filter proteins with transmembrane domains it uses TMHMM2.0. (If the signal peptide contains a TM domain, it will still pass this stage). Next, the pipeline identifies N-terminal sorting peptides *via* TargetP. Lastly, using WolfPsort, it identifies sequences likely to be in the extracellular compartment.

### Statistical Analysis

For the transcriptome profiling, from the output of Cuffdiff program all genes showing p-value < 0.05 (two-sided, adjusted by Benjamini-Hochberg method) were further examined for differential expression and functional processes.

Statistical significance was attributed to p < 0.05 (two-sided) and FDR < 0.05 where applicable. For statistical analysis of pathway analysis, Fisher’s exact test and Bonferroni’s correction for multiple comparisons were applied. GraphPad Prism was used for statistical analyses of the physiological data which are presented as means ± SE.

For human aerobic capacity analysis, we ran Pearson correlation for gene expression of human exercise transcriptome and corresponding mitochondrial respiration using cor.test function in R programming language. From the ouput, genes with correlation p-value < 0.05 (two-sided) were intersected with DEGs in rat aerobic capacity dataset (HCR-LCR) to check the directionality of change. Genes changing in the same direction were reported.

For human exercise response analysis, after sorting the response rates of participants based on the change in mitochondrial respiration, we determined the DEGs for pre- and post-training states of high and low responders. Overlap of DEGs with same directionality of change between humans and rats were selected for analysis.

## Results

### Muscle Transcriptional Signature of Aerobic Exercise Capacity Highlights Enhanced Vascularization and Oxygenation

**Figure 1:**
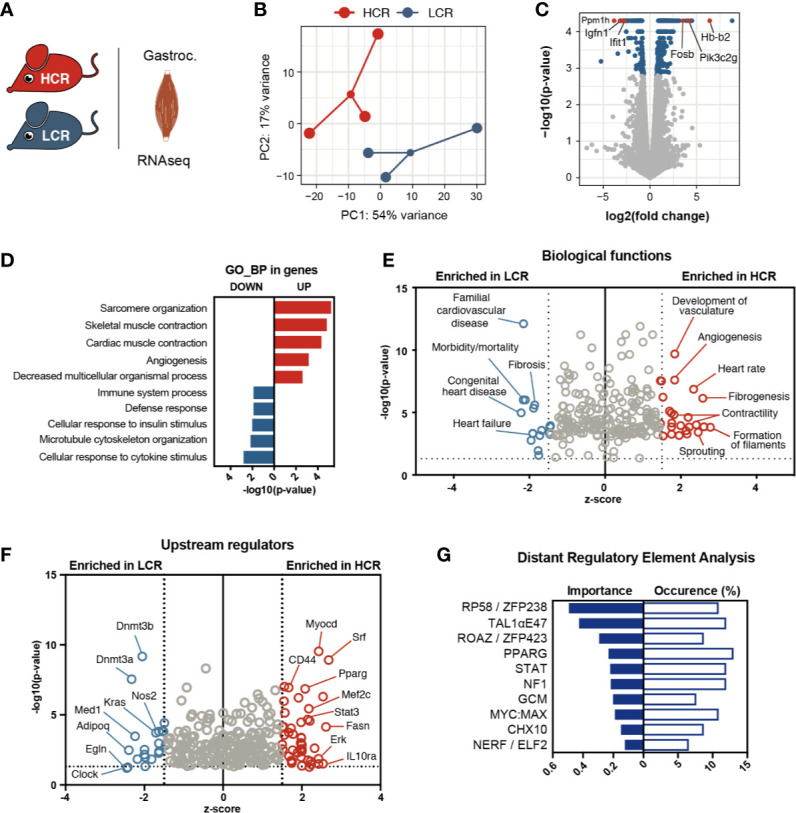
Transcriptional signature of aerobic exercise capacity in skeletal muscle. **(A)** Comparison of transcriptional profiles with RNA-sequencing of gastrocnemius muscles from High Capacity Runner (HCR) and Low Capacity Runner (LCR) rats. **(B)** Principle component analysis of HCR and LCR groups (n = 3). **(C)** Volcano plot showing gene expression analysis, genes with FDR < 0.05 shown in blue, and highlighted genes in red. **(D)** PANTHER gene ontology analysis showing top 5 biological processes (GO_BP) enriched in upregulated genes (red) and top 5 terms enriched in downregulated genes (blue). Hierarchically connected, largely redundant GO terms are represented by the smallest set (Fold enrichment > 5, p < 0.05, Bonferroni correction for multiple comparison). **(E, F)** Ingenuity pathway analysis for biological functions and upstream regulators (input FDR<0.05, z-score cut-off 1.5 on each side of the axis, only data with p < 0.05 plotted). **(G)** Distant regulatory element analysis of the differentially regulated genes (set to target top 3 ECRs + promoter ECRs against 5000 random background).

To investigate the molecular mechanisms underlying intrinsic aerobic capacity, we used skeletal muscle from male rats selectively bred based on their running capacity (**Figure 1A**). Without any exercise training, the HCR group largely outperformed the LCR group on a maximal treadmill performance test (**Supplementary Figure 1A**). To identify molecular pathways associated with this intrinsic difference in exercise capacity, we performed global analysis of gene expression by massively parallel RNA-sequencing on gastrocnemius muscles from these animals (**Figure 1A**). We observed a similar range and distribution of reads across all samples confirming a comparable transcriptomic coverage. Despite the heterogeneous nature of these models, individual transcriptomes clustered according to exercise capacity (**Figure 1B**). By comparing the skeletal muscle transcriptomes of both groups, we identified 434 differentially expressed genes (DEGs), out of which 257 were increased in HCR and 177 were elevated in LCR (fold change > 1.5, q < 0.05) (**Figure 1C**). Among the top 10 transcripts with higher expression in HCR were genes linked to metabolism (*Pik3c2g* and *Mup4*), cell proliferation and differentiation (*Fam163a*, *Fosb*, and *Cyp2J4*), and regulation of blood vessel function and oxygen supply (*Msln*, *Cyp2J4*, and *Hb-b2*) (**Figure 1C** and **Supplementary Figure 1B**). Conversely, several of the top genes with higher expression in LCR muscle (i.e., 10 lowest in this analysis), were related to myogenesis and muscle regeneration, including *Tmem8c* (also known as Myomaker), *Ranbp3l*, and *Ppm1h*. *Igfn1*, a negative regulator of protein synthesis, was highly expressed in LCR. Also, *Ifit1* expression was higher in LCRs, which encodes for a component of interferon-induced protein complex that was previously linked to obesity-related inflammation (27).

To have a global view of the muscle gene programs associated with enhanced intrinsic capacity, we performed gene ontology (GO) analysis on the DEGs. The transcripts increased in HCR muscle revealed enrichment for genes associated with muscle structure, contractility and vascularization (**Figure 1D**). On the other hand, immune response-related genes were overrepresented within the LCR transcriptome. In parallel, we used Ingenuity Pathway Analysis (IPA) to further uncover pathways and functions that may contribute to the divergent phenotypes of these models (**Figure 1E**). Based on the differential gene expression, IPA predicts whether a specific biological function is activated (positive z-score) or inhibited (negative z-score). In this context, an inhibition of a function for HCR means an activation for LCR. Consistent with the GO analysis, angiogenesis and contractility related functions were heavily represented among the biological functions that were predicted to be activated in HCR muscles. One interesting biological function highlighted in the IPA analysis was fibrogenesis, representing genes associated with extracellular matrix remodelling (e.g., *Plat*, *Serpine1*, *Thbs1*, and *F2*) and that were elevated in high capacity runner transcriptome (**Supplementary Figure 1C**). Biological functions activated in the LCR muscle transcriptome involved cardiovascular disease terms and mortality reflecting the metabolic problems reported in these animals (28).

To explore how these gene programs are orchestrated, we interrogated the DEG sets for putative upstream regulators. Searching for coordinated expression patterns in the DEGs against co-expression databases, IPA retrieved several transcription factors known to be important for muscle function (Mef2c, PPARγ, SRF, and STAT3) as activated in HCR (**Figure 1F**). On the LCR side, the DNA methylases Dnmt3a and Dnmt3b scored high among the potential regulators. Although with less significance, we also spotted regulators that may play a role in oxygenation state and injury recovery (Nos2 and Egln). Another way to identify putative upstream regulators, is to scan the close vicinity of co-regulated genes for known transcription factor binding motifs (TFBM). To that end, we used DiRE (distant regulatory elements of co-regulated genes) (25) to analyze the regulatory regions of DEGs (**Figure 1G**). With this approach, we observed again an association of STAT and PPARγ motifs with HCR-related genes and identified additional TFs, some of which are recently shown to modulate myogenesis (Zfp238) (29) and muscle regeneration (Zfp423) (30).

### Reduced Response to Endurance Training Is Associated With Inflammatory Gene Signature and Circadian Rhythm Regulation

**Figure 2:**
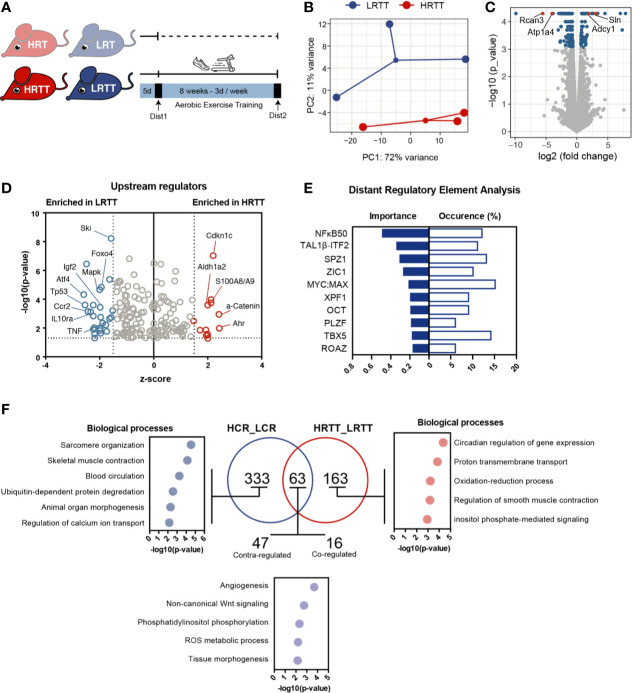
Adaptive exercise response transcriptional profile in skeletal muscle. **(A)** High and low responder rats were trained on a treadmill for 8 weeks generating low and high responder to training-trained rats (LRTT and HRTT). **(B)** Principle component analysis of LRTT and HRTT skeletal muscle transcriptomes (n = 3) **(C)** Volcano plot showing gene expression analysis, genes with FDR < 0.05 shown in blue, and highlighted genes in red. **(D)** Ingenuity Pathway Analysis showing predicted upstream regulators (input FDR < 0.05, z-score cut-off of 1.5 on each side of the axis, only data with p < 0.05 plotted). **(E)** Transcriptional factors for the differentially regulated genes predicted by distant regulatory element analysis (set to target top 3 ECRs + promoter ECRs against 5000 random background). **(F)** Venn diagram comparison of high capacity and high response transcriptional profiles and Panther gene ontology analysis for biological processes for corresponding gene signatures. Hierarchically sorted GO terms (Fold enrichment >10, p < 0.05, Bonferoni correction for multiple testing) were ranked for the lowest p-value, and top 5 were plotted.

The large inter-individual variation in response to exercise training poses considerable challenges in the design of standardized exercise interventions. To map the exercise response transcriptome of skeletal muscle, we used the selectively bred HRT-LRT model system and trained them on a treadmill for 8 weeks, generating the HRT-Trained (HRTT) and LRT-Trained (LRTT) groups (**Figure 2A** and **Supplementary Figures 2A–C**). A separate cohort of untrained HRT and LRT animals underwent a maximal running capacity test before and after the 8 weeks training period. While the pre-training performance of HRTs and LRTs were the same, HRTTs significantly outperformed the LRTTs in the post-trained condition (**Supplementary Figures 2B–C**). Reflecting this, the HRTT and LRTT transcriptomes clustered separately (**Figure 2B**) with 255 differentially expressed genes (fold change > 1.5, FDR < 0.05). Of these, 68 were enriched in HRTT and 187 were decreased in HRTT (i.e., enriched in LRTT). Among the top increased transcripts in HRTT muscles were some genes involved in training-induced pathways, such as calcium signalling (*Sln* and *Adcy1*) but also some novel ones *(Kcna10* and *Lgi3*). (**Figure 2C** and **Supplementary Figure 2D**). Interestingly, Lgi3 has been reported to activate Akt although in other cell types (31). Top LRTT enriched transcripts included negative regulators of calcineurin signalling (*Rcan3*), positive regulators of NMJ function (*Wnt16* and *VAT1l*), and slow twitch fibres (*Myh6*). To investigate pathways and functions that potentially contribute to training response, we explored enriched gene sets with GO analysis and biological functions (**Supplementary Figures 2E, F**). High response transcriptome revealed a blood pressure related signature while low response signature included circadian regulation (specifically *Per1*, *Bhlhe40*, *Ciart*, *Db*p expression induced, and Nfil3 repressed) and was overall more diversified. When we looked at the potential upstream regulators, transcription factors Foxo4 and Atf4 known for their roles in skeletal muscle atrophy (32–35), were among the predicted factors whose targets were enriched in the LRTT transcriptome (**Figure 2D**). This is in line with the reports showing lack of response to strength training in LRTTs (36). In addition, immune response-related factors (Ccr2, TNF, IL10ra, and p53) were also among the activating factors for the LRTT muscle indicating a damage response. In support of this, a separate search for potential regulators revealed an enrichment of the TFBM for NFkB50 (p50-p50 homodimer), a known transcriptional repressor and regulator of immune and inflammatory responses (**Figure 2E**).

Comparing the individual effects of the intrinsic and acquired components of exercise capacity could prove useful to investigate common and unique underlying mechanisms. A Venn diagram of the transcriptional profiles revealed the unique gene sets to each component (**Figure 2F**). The intrinsic capacity unique transcripts (n = 333) reflects a predominant muscle function signature enriched with sarcomere organization, skeletal muscle contraction, and regulation of Ca^++^ ion transport biological processes. On the other hand, the signature for acquired capacity (n = 163) displays a more fragmented signature with associations to circadian regulation, proton transmembrane transport and redox process. The 63 shared transcripts were mostly associated with angiogenesis, ROS metabolic process, and Wnt signalling, all of which are known exercise induced processes.

Since the HRT and LRT training response diverges, we next considered if differences in exercise-induced changes in their transcriptional profiles could uncover candidate genes linked to enabling or inhibiting such exercise adaptations. To explore this angle, we compared each group in the post-trained state to their corresponding pre-training state (**Supplementary Figure 2G**). Most training-induced genes common to low and high exercise responders were regulated in the same direction. The unique genes for low training response (n = 193) were enriched in GO terms related to immune response (inflammation and interferon responses and receptor internalization) and effects of exercise (angiogenesis, synaptic vesicle transport, and ROS metabolic process). Conversely, the gene signature unique to high response was enriched in processes concerning ion transport, Ca^++^ signalling, and oxidative respiration.

### The Predicted Muscle Secretomes of Exercise Capacity and Response Highlight Lipid Metabolism and Extracellular Matrix Remodelling

**Figure 3:**
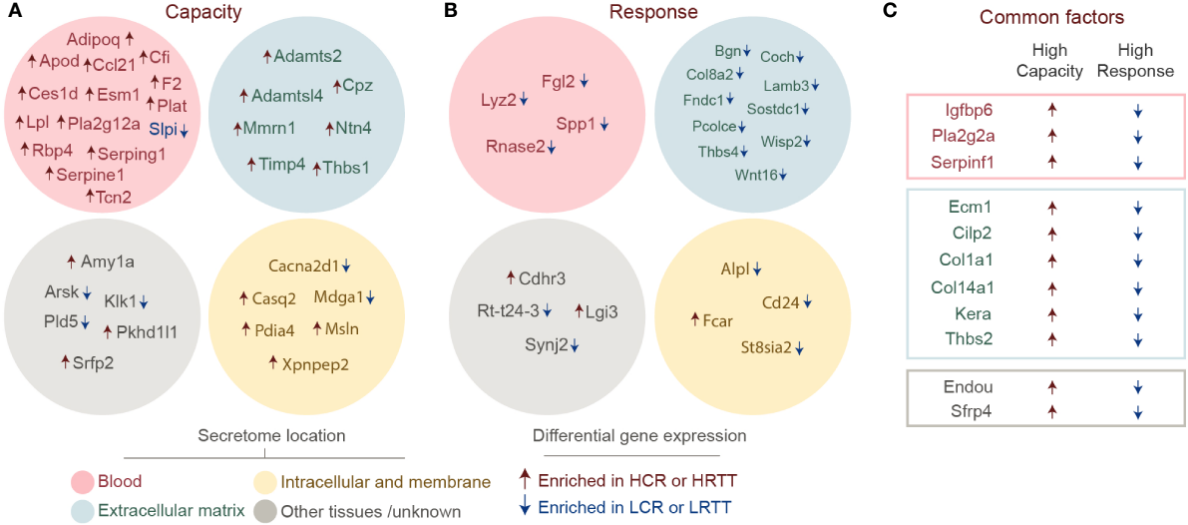
Secretome prediction for high exercise capacity and high response to exercise. Differentially expressed genes for high capacity and high response were used to search for predicted secreted factors. Factors unique to **(A)** high capacity and **(B)** high response muscle secretomes are shown divided to locations based on data from Human Protein Atlas: blood (pink), extracellular matrix (blue), intracellular and membrane (yellow), and other tissues/unknown locations (gray). Upregulated transcripts are marked with a red arrow and a blue arrow marks the downregulated transcripts. **(C)** Factors that are common to high capacity and high response muscle profiles are shown in boxes with the same color-code (blood in red, extracellular matrix in blue, and other in gray).

Muscle-derived secreted factors play an important role in mediating the exercise-associated local and systemic adaptations (37). We used the transcriptomics data to identify known or novel myokines involved in exercise capacity or response phenotypes (**Figure 3**). To this end, transcriptome profiles were processed through a well-established pipeline for protein sequence-based prediction of putative secreted factors (26). The human secretome and membrane proteome resource compiled by the Human Protein Atlas (HPA) initiative (38) allowing us to determine the location of each candidate molecule (**Figures 3A–C**). Based on the capacity and response transcriptomes, we were able to predict some known secreted factors (Lpl, Plat, Adipoq, Lyz2, Igfbp6, and Tcn2) and some putative ones. Interestingly, a considerable fraction of the predicted capacity secretome is classified as potentially secreted to blood. Nearly all the corresponding transcripts were significantly increased in the HCR transcriptome (**Figure 3A**). Among these, members of the plasmin system *(*F2/Thrombin, Plat, Serping1, and Serpinf), lipid metabolism (ApoD, Adipoq, Ces1, Lpl, and Pla2g12a) and immune function (Ccl21, Cfi, and Slpi) stand out. Notably, nearly all predicted factors for response profile were enriched in LRTTs (reduced in HRTTs). The largest fraction of the predicted secretome profile for exercise response was categorized to ECM as the HPA secretome localization, and included Wnt signalling related factors (Wisp2, Wnt16, and Sostdc) (**Figure 3B**). Interestingly, the shared factors between the two profiles involved proteins related to ECM remodelling (Col1a1, Col14a1, Ecm1, Thbs2, Cilp2, and Serpinf1), insulin sensitivity, and metabolism (Igfbp6 and Pla2g2a) (39) (**Figure 3C**).

### Gene Networks of Exercise Capacity and Response in Humans

**Figure 4:**
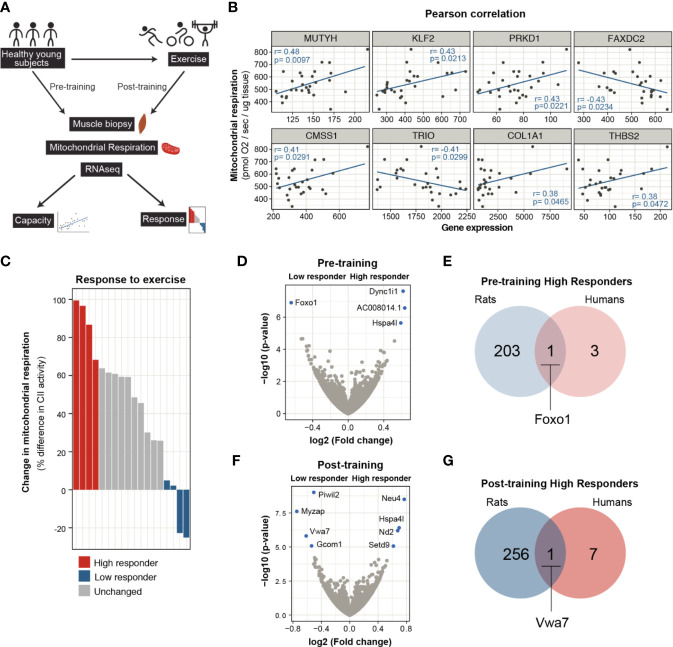
Molecular mediators of intrinsic capacity and response to exercise in humans. **(A)** Scheme showing the dataset and workflow used for the analysis using data from Robinson et. al. **(B)** Pearson correlation analysis of pre-training skeletal muscle mitochondrial respiration measurement and gene expression levels for healthy, young adults (n = 29, p < 0.05, only samples with normal distribution were plotted). **(C)** The subjects are ranked based on change in mitochondrial respiration after aerobic exercise training. Top 4 individuals are designated as high responders (red) and bottom 4 subjects are grouped as low responders (blue). Skeletal muscle transcriptomes of high and low responder subjects were compared by re-analysing RNA-seq data. **(D)** Comparison of pre-training transcriptome profiles is shown in volcano plot (n = 4), transcripts with FDR < 0.05 shown in blue. **(E)** Venn diagram comparison of pre-trained muscle transcriptomes of high responder rats and humans. **(F)** Volcano plot for differentially expressed genes between post-training skeletal muscle transcriptomes of high and low responder humans. **(G)** Post-trained transcriptome comparison for high responder rats and humans.

Next, we sought to determine the molecular factors that play into an individual’s aerobic capacity and their potential to improve endurance performance in a similar way to the rat models we used for this study. For this, we used the RNA-seq datasets from Robinson et al. (20) to find correlations between relative gene expression levels, exercise capacity and response (**Figure 4A**). In that study, healthy young and older participants were exposed to different modalities of exercise and the multiple-omics approach aimed to find molecular transducers of exercise benefits in skeletal muscle.

Using the pre-training muscle mitochondrial respiration measurements as a functional indicator of intrinsic aerobic capacity and RNA-seq from the same volunteers (**Supplementary Table 1**), we looked for correlations between these two measures. From this analysis, we identified 574 genes that positively correlated with muscle mitochondrial respiration values. Within this group, genes fell into biological processes related to muscle regeneration (most notably notch signalling), metabolism, and calcium homeostasis. A smaller number of genes correlated negatively with mitochondrial function and grouped under Mitochodrial RNA processing and Regulation of autophagy, among other (**Figure 4B** and **Supplementary Figure 3A**). From the set of genes that showed a correlation with mitochondrial respiration (either positive or negative), we could find 16 amongst the HCR/LCR DEGs (**Supplementary Table 2**). Eight of these genes were regulated in the same direction in both humans and rats (**Figure 4B** and **Supplementary Table 2**). *MUTYH*, encoding for an enzyme called MYH glycosylase involved in the repair of oxidative DNA damage, showed the highest correlation estimate. In addition, transcript levels of the transcription factor Kruppel like factor 2 (*KLF2*) and Protein Kinase 1D (*PRKD1/*PKD1) also showed a positive correlation with mitochondrial function and the capacity for aerobic exercise. Notably, a cluster of correlated genes (*TRIO*, *COL1A1*, and *THBS2*) hinted toward a role for ECM and cytoskeleton reorganization in determining exercise capacity.

To investigate genes and networks associated with the adaptability to exercise, we used the change in muscle mitochondrial respiration after the 12-week training program as an indicator for the response to exercise. Participants were ranked according to the change in mitochondrial respiration and grouped as high and low responders (**Figure 4C** and **Supplementary Figure 3B**). By comparing the transcriptional profiles of these groups at their pre-training states, we identified four genes differentially expressed in individuals highly responsive to exercise (*DYNC1I1*, *HSPA4L*, *AC008014.1*, and *FOXO1*) (**Figure 4D**). When cross-compared to the transcriptional signature of high responder rats at their pre-training state, *FOXO1* emerges as the common factor according to this model for adaptability to exercise (**Figure 4E**). At the post-training state, the differential expression analysis of the high and low responder individuals yielded eight genes, four of which had increased transcripts (*NEU4*, *HSPA4L*, *ND2*, and *SETD9*) and the other four decreased (*PIWIL2*, *MYZAP*, *VWA7*, and *GCOM1*) (**Figure 4F**). By comparing the post-training DEGs to the trained high responder rat transcriptional profile and identified Vwa7 as a shared factor (**Figure 4G**). Vwa7 is a ubiquitously expressed gene encoding a putatively secreted protein on unknown function.

## Discussion

The interplay between intrinsic and acquired exercise capacities, both of which have high inter-individual variability (7, 40), represents an obstacle to identifying the exact mechanisms connecting aerobic exercise capacity and human health. By using rat and human gene expression data associated with a specific exercise-related functional measure, we uncovered muscle transcriptional signatures underlying the inherent aerobic capacity and responsiveness to training in both species.

The transcriptional profile of HCR muscle was strongly associated with pathways that regulate vascularization and oxygen transport. These molecular signatures echo previously reported functional data from the same model showing enhanced oxygen uptake and higher capillary density in HCR muscle (11–13, 41), and both are well-known adaptations to aerobic exercise both in rodents and humans (42). In contrast, gene expression patterns in LCR muscle displayed an enriched profile for immune response-related functions. Importantly, there is an established link between physical inactivity and chronic low-grade inflammation. In fact, inactivity is linked to increased morbidity and mortality due to chronic pathologies (2, 43). Interestingly, the muscle transcriptome of LCRs associates with biological functions related to cardiovascular disease, further reflecting their poor health condition as previously documented (28, 44–48). Remarkably, the same analysis also pointed out a curious association to morbidity/mortality. Taken together, all of these suggest that the transcriptional signatures we determined are reflective of some verified functional outputs and therefore can be used to gain new insights on the mechanisms critical to aerobic exercise capacity.

Transcriptional profiles can be useful gateways to explore how particular gene programs are regulated in a concerted way. Identifying potential transcription factors and coregulators associated to the extreme capacity and response phenotypes could give us clues about the drivers of divergent mechanisms. One such example were the DNA methylases *Dnmt3a* and *Dnmt3b*, whose activity was predicted to be reduced in the HCRs (activated in LCRs). Interestingly, Dnmt3b activity has been previously shown to limit bioenergetic adaptations to exercise in muscle, by increasing promotor methylation of specific genes (49). DNA methylation has been extensively linked to transgenerational inheritance of different traits, including response to exercise (50) and could be part of a possible regulatory mechanism to transmit exercise capacity to the next generations.

Our analysis also suggested some TFs as potential mediators of the aerobic capacity and response. Spatiotemporal expression of TFs plays a critical role in muscle development, metabolism and in its adaptations to exercise (3, 4). Based on our data, one candidate regulator of exercise capacity is RP58 (also known as Zfp238), which has been identified to modulate developmental myogenesis (29). Another is ROAZ (Zfp423), which was recently implicated for its role in muscle regeneration by promoting expansion of satellite cells (30), which are important for muscle fiber repair/remodelling in response to exercise (51).

Identifying the determinants and mediators of the acquired capacity holds a great promise for customization of exercise programs based on individual trainability. The transcriptional signature for exercise response in skeletal muscle may appear less obvious compared to the intrinsic capacity signature. While there is significant evidence that the HCR phenotype is mainly driven by skeletal muscle (11, 13), other organs or systems may play a greater role in the trainability phenotype. Regular exercise requires sustaining repeated stress to the body, including cardiovascular system and non-contractile, mechanical components such as joints and tendons (42). In addition, it is widely known that central nervous system contributes to metabolic regulation and may play a role in exercise capacity and trainability as it governs motor function and behavioral components (such as motivation). Any of these, or other factors, could also be limiting for the adaptation to exercise in low responders. In fact, training activated a larger gene program in low responders than in high responders. It is tempting to speculate that in low responders there might be an inhibitory signaling network hampering training adaptations. Emerging evidence indicates elevated inflammatory signaling and increased metabolic dysfunction in LRTT rats (52). Interestingly, circadian regulation of gene expression stands out in pathway analysis of the response signature when compared to the capacity transcriptome. Exercise is known to synchronize to the biological clock and there is evidence that the timing of the day affects exercise performance (53). Finally, exercise intensity has been suggested to be a main driver of response in humans (54). To reduce this possibly confounding effect we used data from a human exercise intervention that includes High Intensity Interval Training (20), which indeed resulted in a VO_2_max and mitochondrial respiration improvement in almost all participants.

Identifying novel muscle-secreted factors could offer new ways to harness some of the benefits of physical activity. Based on the muscle transcriptome, the corresponding secretome profile of HCRs indicates a higher presence of factors secreted to the blood. It is tempting to speculate that HCR muscles are readily equipped to send necessary circulating factors to aid high aerobic performance such as those involved in lipid metabolism and improved circulation. Based on the muscle secretome predictions, ApoD together with Lpl and Ces1d may facilitate fatty acid uptake to the muscle. This may contribute to the previously reported efficient fuel utilization and energy metabolism in HCR muscles (55). On the other hand, the responder profile suggests an increase in secreted factors primarily to the extracellular matrix. LRTs may be signaling for ECM remodeling due to the failure of coping with the prolonged stress of exercise training. This goes in line with previous studies of networks regulated in response to exercise both in rats and in humans (52, 56). Expanding our knowledge about muscle-secreted factors regulated by the intrinsic or adaptive exercise response could pave the way for tailored exercise-based therapies.

The premise of the outbred, genetically heterogeneous rat models for exercise capacity is partly to reflect the complexity of metabolic diseases and the diversity among humans. We utilized a human dataset from Robinson et al. (20) to search for a bridge between the transcriptome profiles of aerobic capacity and trainability in rats and humans. Despite the clear inter-species differences and the small cohort, we were able to identify 8 genes whose expression correlated with intrinsic capacity in humans and shared with rat exercise capacity transcriptome. Among these, Prkd1 (PKD1) expression positively correlated with aerobic exercise capacity in humans. PKD1 is a class II HDAC kinase shown to promote slow-twitch fatigue-resistant phenotype in skeletal muscle (57). This is intriguing because inherited factors that affect aerobic exercise capacity could involve epigenetic modifications. In fact, accumulating evidence suggests that epigenetic marks can mediate transmission of exercise and diet effects to the next generation (50). Comparison of response signatures pointed out *Foxo1* as a common factor in both rats and humans. Foxo1 is a transcription factor known to be induced in settings of skeletal muscle atrophy promoting autophagy and ubiquitin-proteosome system (32). Regarding the exercise response, increased Foxo1 levels in low responders may indicate an elevated protein degradation to clear the damaged proteins as a result of prolonged training stress.

This study provides a foundation for elucidating the molecular map for exercise capacity and response. Among the different transcriptional signatures linked to the exercise performance-related measures we analyzed, angiogenesis and oxygen delivery emerge as main drivers of intrinsic capacity. Conversely, pro-inflammatory signaling is linked to lower adaptation to training. A better understanding of the underlying mechanisms could ultimately pave the way for personalized training programs that optimize health outcomes based on individual needs.

## Limitations

In this study, we utilized unique rodent models for exercise capacity to identify sets of genes/transcripts that contribute to intrinsic and adaptive components of exercise capacity. Of note, the breeding strategy used to generate these rat models, may lead to the fixation of gain- or loss-of-function genetic mutations, which we did not investigate. During the analysis, we noted a high degree of variability which can be explained with the out-bred nature of these models. Further studies with larger cohorts or targeted approaches could investigate the potential links highlighted in this study and shed light into the underlying mechanisms. Our exploratory approach comparing human and rodent data-sets does not indicate causality and serves as a clue for further evaluation. Lastly, body weight can influence aerobic running capacity and also plays a substantial role in the emergence of metabolic and cardiovascular diseases. Many generations of selective breeding for aerobic capacity led to a significant correlation with body weight in the HCR and LCR lines. However, the body weight increase in LCR and decrease in HCR did not further diverge after generation 13 and was reported to be stabilized at 0.7–0.8 fold by generation 28 (9). Furthermore, taking body weight and generation as predictors of aerobic capacity in multiple regression analysis, Wisloff et al. (28) revealed that even though body weight accounts for some variation in the running distance (7% for females, 14–20% for males), the majority of the variation is due to other factors.

## Data Availability Statement

RNAseq datasets generated for this study can be found in NCBI Gene Expression Omnibus (GEO) (https://www.ncbi.nlm.nih.gov/geo/) under accession number GSE155230. Robinson et al. RNAseq datasets can be accessed in GEO with GSE97084 accession number. The rat models for low and high intrinsic exercise capacity and low and high exercise response to training are maintained as an international resource with support from the Department of Physiology & Pharmacology, The University of Toledo College of Medicine, Toledo, OH. Contact LK (Lauren.Koch2@UToledo.edu) or SB (brittons@umich.edu) for information on the rat models.

## Ethics Statement

The studies involving human participants were reviewed and approved by Mayo Clinic Institutional Review Board and registered under Clinical Trials #NCT01477164 and # NCT01738568 (clinicaltrials.gov). The patients/participants provided their written informed consent to participate in this study. The animal study was reviewed and approved by University Committee of Use and Care of Animals at the University of Michigan.

## Author Contributions

JLR, PRJ, and YK designed the study. LGK, SLB, HW, KSN, MMR, and JLR provided the resources. IC, JZ, PRJ, and YK performed the data analysis. YK and JLR wrote the initial draft of the manuscript. All authors contributed to the article and approved the submitted version.

## Funding

This work was supported by the Swedish Research Council (2016-00785) and Novo Nordisk Foundation (NNF19OC0054132) to JR., the Wenner-Gren Foundations (Sweden) (UPD2017-0175), FAPESP (Brazil) (14/26797-5) to PJ, The Swedish Society for Medical Research to IC, the National Center for Advancing Translational Sciences (UL1TR000135) to KSN, and the National Institutes of Health (USA) (T32DK7352 to MR and R01AG09531 to KSN and P40ODO21331 to LK and SB).

## Conflict of Interest

JR is a consultant for Bayer AG.

The remaining authors declare that the research was conducted in the absence of any commercial or financial relationships that could be construed as a potential conflict of interest.
