# Supplementary material for: Comparative Analysis of Skeletal Muscle Transcriptional Signatures Associated With Aerobic Exercise Capacity or Response to Training in Humans and Rats
Source: Front Endocrinol (Lausanne). 2020 Oct 26;11:591476. doi: 10.3389/fendo.2020.591476 (PMC7649134; doi:10.3389/fendo.2020.591476)
Supplement: Supplementary file 1 [file DataSheet_1.pdf]

A

	LCR (n=3)	HCR (n=3)
Body weight (g)*	342,9 ± 4,58	211,44 ± 9,27
Time to exhasution (min)**	11,5 ± 0,142	83,31 ± 1,124
Distance (m)**	141,5 ± 2,28	2527 ± 57,54
Total work (J)**	123,1 ± 1,11	1358 ± 83,45
Best speed**	15 ± 0	51 ± 0,58

*p < 0,0002, **p<0,0001

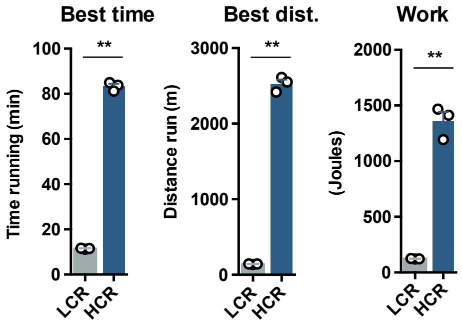

B

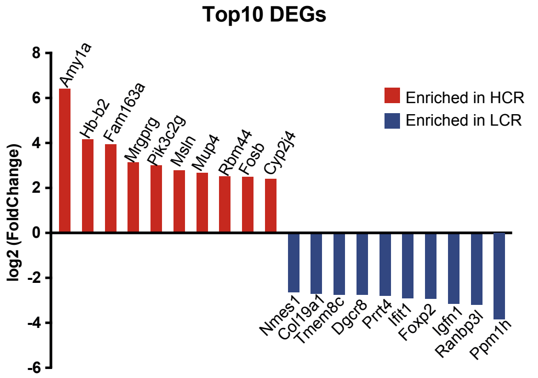

C

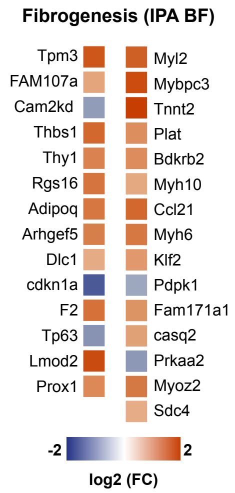

Supplementary Figure 1

(**A**) Maximal capacity treadmill running performance of generation 33 HCR and LCR groups (males, n=3, unpaired t-test with two-tailed p-value, 99% CI) (**B**) Top 10 differentially expressed genes for HCR and LCR groups. (**C**) Heatmap of differentially expressed genes that belong to Fibrogenesis biological function of IPA (Fold change p adjusted < 0.05).

A

	LRT (n=3) †	LRTT (n=3)	HRT (n=3) †	HRTT (n=3)
**Body weight (g)**				
Before training	323 ± 14,5	288 ± 14,05	342 ± 21,18	281 ± 25,71
After training	396 ± 15,72	324 ± 22,33	422 ± 30,37	336 ± 25,09
Δ BW (g)	73 ± 3,93	34 ± 5,04	69 ± 7,37	53 ± 9,15
% BW	22 ± 0,03	11 ± 0,02	22 ± 0,04	19 ± 0,37
**Distance (meters)**				
Before training	620 ± 161	546 ± 24	502 ± 33,44	522 ± 56,95
After training*	351 ± 12,82	459 ± 30,09	343 ± 42,06	806 ± 114,66
Training Response (Δ distance)	-239 ± 83,80	-67 ± 10,33	-106 ± 46,48	284 ± 18,39
% dist**	- 36 ± 0,02	- 16 ± 0,02	-32 ± 0,119	55 ± 0,08

† not trained but evaluated at the end of 8w taining period, *p < 0,0002, ** p<0,0001

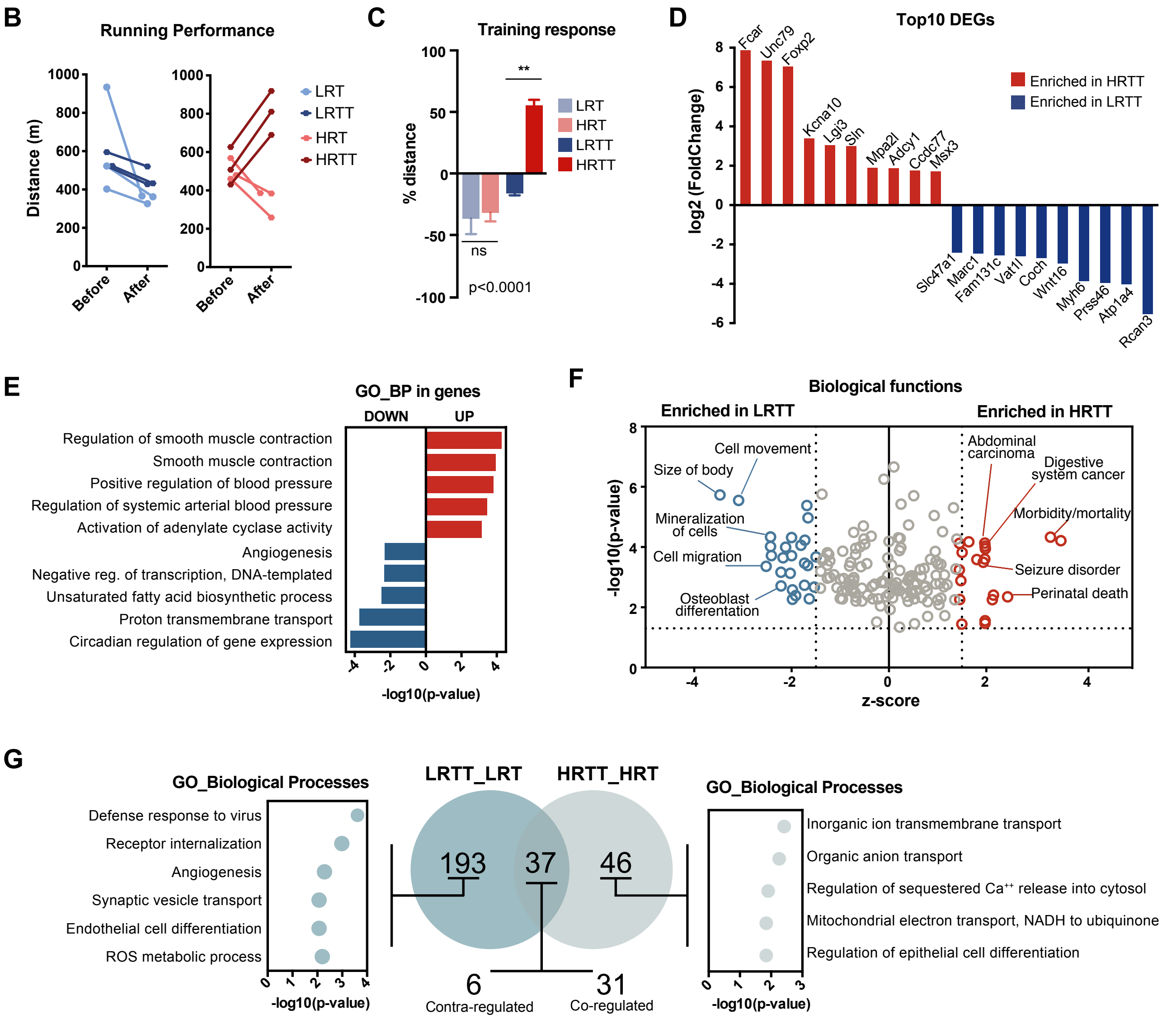

Supplementary Figure 2

(**A**) Physiological data of the of generation 15 responder lines (**B**) Maximal capacity treadmill running performance before and after the 8 week training period. (**C**) Training response plotted as per cent improvement of distance (DIST2-DIST1) and as data ± the standard error of mean (SEM), (males, n=3), ordinary two-way ANOVA with Bonferroni's multiple comparison test (99% CI). (**B**) Top 10 differentially expressed genes for HRTT and LRTT groups, q < 0,05 (**C**) PANTHER GO_Slim Biological Processes enriched in the response to training transcriptome. (**D**) IPA biological functions with the exercise response transcriptome (input FDR<0.05, z-score cut-off 1.5 on each side of the axis, only data with p<0.05 plotted). (**E**) Venn diagram comparison of low response and high response transcriptional profiles. Panther gene ontology analysis for biological processes for unique gene signatures. GO terms for biological processes (p<0.05, two-tailed) Bonferroni's correction for multiple comparison) were sorted for the lowest p-value, and top 5 were plotted.

Supplementary Table 1 | Mitochondrial respiration measurements from human participants pre- and post-training.

Run_ID	Gsm_ID	ID	Age	Exercise module	Sample collection	pmol O2/sec/ ug tissue
SRR5381159	GSM2551325	10C	Young	Combined	Post	711,47775
SRR5381158	GSM2551324	10B	Young	Combined	Pre	442,31725
SRR5381161	GSM2551327	11B	Young	Endurance	post	805,61945
SRR5381160	GSM2551326	11A	Young	Endurance	pre	431,5635
SRR5381163	GSM2551329	13B	Young	Resistance	post	303,1689
SRR5381162	GSM2551328	13A	Young	Resistance	pre	390,7143
SRR5381165	GSM2551331	14C	Young	Combined	post	809,18
SRR5381164	GSM2551330	14B	Young	Combined	pre	643,48635
SRR5381167	GSM2551333	15B	Young	Resistance	post	592,9212
SRR5381166	GSM2551332	15A	Young	Resistance	pre	515,3973
SRR5381169	GSM2551335	16B	Young	Resistance	post	374,3844
SRR5381168	GSM2551334	16A	Young	Resistance	pre	441,8533
SRR5381171	GSM2551337	17B	Young	Endurance	post	832,6496
SRR5381170	GSM2551336	17A	Young	Endurance	pre	494,96415
SRR5381173	GSM2551339	18B	Young	Endurance	post	617,2434
SRR5381172	GSM2551338	18A	Young	Endurance	pre	604,11245
SRR5381175	GSM2551341	19B	Young	Resistance	post	651,18015
SRR5381174	GSM2551340	19A	Young	Resistance	pre	479,74625
SRR5381181	GSM2551347	21C	Young	Combined	post	415,56535
SRR5381180	GSM2551346	21B	Young	Combined	pre	537,8442
SRR5381187	GSM2551353	24B	Young	Endurance	post	1025,7368
SRR5381186	GSM2551352	24A	Young	Endurance	pre	643,9444
SRR5381189	GSM2551355	25B	Young	Resistance	post	912,6478
SRR5381188	GSM2551354	25A	Young	Resistance	pre	495,07825
SRR5381191	GSM2551357	26B	Young	Endurance	post	763,0602
SRR5381190	GSM2551356	26A	Young	Endurance	pre	524,36755
SRR5381192	GSM2551358	27B	Young	Combined	pre	566,97105
SRR5381196	GSM2551362	29B	Young	Resistance	post	590,5742
SRR5381195	GSM2551361	29A	Young	Resistance	pre	555,7444
SRR5381198	GSM2551364	2C	Young	Combined	post	637,3855
SRR5381197	GSM2551363	2B	Young	Combined	pre	489,9474
SRR5381200	GSM2551366	30B	Young	Resistance	post	450,4095
SRR5381199	GSM2551365	30A	Young	Resistance	pre	607,3589
SRR5381202	GSM2551368	31C	Young	Combined	post	519,8583
SRR5381201	GSM2551367	31B	Young	Combined	pre	495,49795
SRR5381204	GSM2551370	32B	Young	Endurance	post	1143,575
SRR5381203	GSM2551369	32A	Young	Endurance	pre	718,345
SRR5381206	GSM2551372	33B	Young	Resistance	post	738,1383
SRR5381205	GSM2551371	33A	Young	Resistance	pre	572,6418
SRR5381210	GSM2551376	35B	Young	Endurance	post	356,02475
SRR5381209	GSM2551375	35A	Young	Endurance	pre	475,1511
SRR5381212	GSM2551378	36C	Young	Combined	post	674,35475
SRR5381211	GSM2551377	36B	Young	Combined	pre	454,0649
SRR5381216	GSM2551382	38B	Young	Resistance	post	697,29665
SRR5381215	GSM2551381	38A	Young	Resistance	pre	822,91385
SRR5381218	GSM2551384	39B	Young	Endurance	post	792,74815
SRR5381217	GSM2551383	39A	Young	Endurance	pre	484,11135
SRR5381220	GSM2551386	3B	Young	Resistance	post	750,58565
SRR5381219	GSM2551385	3A	Young	Resistance	pre	630,5697
SRR5381222	GSM2551388	40B	Young	Endurance	post	902,46065
SRR5381221	GSM2551387	40A	Young	Endurance	pre	716,3848
SRR5381224	GSM2551390	41C	Young	Combined	post	1021,91395
SRR5381223	GSM2551389	41B	Young	Combined	pre	632,69815
SRR5381254	GSM2551420	6B	Young	Endurance	post	678,11495
SRR5381253	GSM2551419	6A	Young	Endurance	pre	340,04805

Supplementary Table 2 | Pearson correlation output for mitochondrial respiration and gene expression in the skeletal muscle of untrained human participants and corresponding gene expression levels of overlapping genes in the skeletal muscle of HCR and LCR rats.

High vs low aerobic capacity human subjects				HCRvsLCR rats				
**ENSEMBL ID**	**Gene symbol**	**Correlation Estimate**	**p_value**	**Gene**	**log2_FC**	**p_value**	**q_value**	**Same direction?**
ENSG00000132781	MUTYH	0,4801	0,0097	mutyh	1,64671	5,00E-05	0,0031	Yes
ENSG00000149043	SYT8	0,4592	0,0140	syt8	1,89491	5,00E-05	0,0031	Yes
ENSG00000127528	KLF2	0,4332	0,0213	klf2	1,00426	5,00E-05	0,0031	Yes
ENSG00000184304	PRKD1	0,4308	0,0221	prkd1	1,04143	0,0012	0,0409	Yes
ENSG00000184220	CMSS1	0,4126	0,0291	cmss1	1,20486	5,00E-05	0,0031	Yes
ENSG00000108821	COL1A1	0,3794	0,0465	col1a1	1,21298	5,00E-05	0,0031	Yes
ENSG00000186340	THBS2	0,3783	0,0472	thbs2	1,10797	5,00E-05	0,0031	Yes
ENSG00000038382	TRIO	-0,4107	0,0299	trio	-0,796355	4,00E-04	0,0176	Yes
ENSG00000170271	FAXDC2	-0,4272	0,0234	faxdc2	-0,771248	0,0012	0,0409	Yes
ENSG00000079215	SLC1A3	-0,4603	0,0137	slc1a3	1,36407	0,00085	0,0313	No
ENSG00000175445	LPL	-0,3983	0,0358	lpl	1,06226	5,00E-04	0,0211	No
ENSG00000188729	OSTN	0,3950	0,0375	ostn	-2,54712	5,00E-05	0,0031	No
ENSG00000205678	TECRL	-0,3895	0,0405	tecrl	1,99759	5,00E-05	0,0031	No
ENSG00000249992	TMEM158	0,3775	0,0476	tmem158	-1,5158	5,00E-05	0,0031	No
ENSG00000172869	DMXL1	0,3772	0,0479	dmxl1	-0,769033	6,00E-04	0,0244	No
ENSG00000138622	HCN4*	0,3942	0,0379	hcn4	1,60971	5,00E-05	0,0031	Yes
ENSG00000130300	PLVAP†	0,3740	0,0499	plvap	0,942577	2,00E-04	0,0101	Yes

*did not follow normal distribution

† p=0.05

A

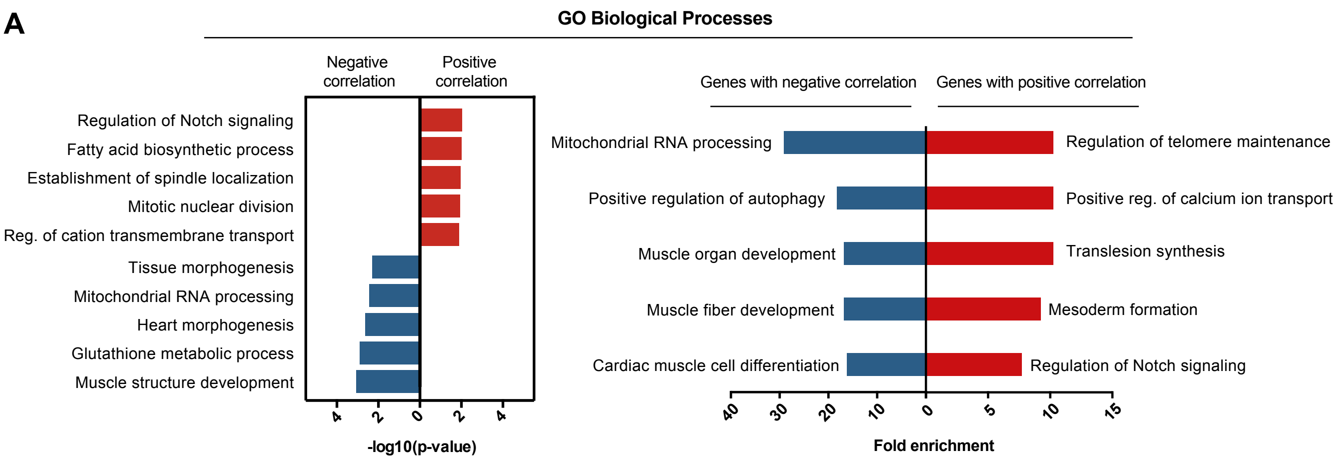

B

ID	Age	Exercise	Sex	Mitochondrial respiration† Pre-training	Mitochondrial respiration† Post-training	% difference	Group
11	Young	Endurance	F	432	806	86.7	Responder
17	Young	Endurance	M	495	833	68.2	Responder
18	Young	Endurance	F	604	617	-2.17	Low responder
21	Young	Combined	F	538	416	-22.7	Low responder
27	Young	Combined	M	567	1115	96.6	Responder
31	Young	Combined	M	495	520	4.92	Low responder
35	Young	Endurance	F	475	356	-25.1	Low responder
6	Young	Endurance	F	340	678	99.4	Responder

† pmol O_2/s/µg tissue

Supplementary Figure 3

(**A**) PANTHER GO Slim biological processes enriched in the genes correlated with mitochondrial respiration humans. Statistically significant GO terms enriched in positively correlated genes (red) and in negatively correlated genes (blue) were sorted for the lowest p-value and most fold enrichment. Top five terms for both conditions were plotted. Hierarchically connected, largely redundant GO terms are represented by the smallest set. (Fold enrichment > 5, p<0.05, Bonferroni correction for multiple comparison). (**B**) Participants ranked according to the change in mitochondrial respiration and grouped as high and low responders for further gene expression analysis.
